# Richmond Academy of Medicine and Surgery

**Published:** 1896-09

**Authors:** Mark W. Peyser

**Affiliations:** Secretary and Reporter


### Society Reports.

---

### RICHMOND ACADEMY OF MEDICINE AND SURGERY.

Regular meeting, August 11th, 1896, Hall of Y. M. C. A. Dr. Landon B. Edwards, President, in the Chair. Dr. Mark W. Peyser, Secretary and Reporter.

### DISLOCATION OF THE ARCH OF THE FOOT.

Dr. J. W. Henson reported a case of dislocation of the arch of the foot. A woman, on June 9th, while crawling along a shed to reach a window, fell a distance of fifteen feet, alighting on her feet, dislocating the arch of the right foot. The foot turned outward like that of a negro, and the internal cuneiform could be felt in the middle of it. She had made a wooden sandal to support the arch. There was no injury elsewhere, except rupture of the internal lateral ligament of the left knee.

Dr. M. D. Hoge, Jr., reported a similar case. Man, age 20, while in a somnambulistic state, climbed out of a fourth story window, falling on his feet. The arch of one foot was completely broken, and it spread out flat—a protuberance being discernible in the middle. He had made a leather wedge which was put in the sole of the shoe, the thick edge inward. The man now walks as well as any one.

### THE ANTI-TUBERCLE SERUM TREATMENT OF CONSUMPTION.

Dr. J. Allison Hodges mentioned his experience with this treatment in several cases, not all of them having been benefited by it, but referred especially to the following case: He was called on May 10th to see Mr. A., aged 42, who was confined to bed—temperature, 102.5°; respiration, 26; great difficulty in breathing, attended with an incessant cough and expectoration, and intermittent pain in right side. On examination, he found consolidation of the lower two-thirds of the right lung, with a suspected abscess forming. The sputum was subjected to microscopic examination, and revealed the presence of tubercle bacilli in large numbers. In response to questions as to the former history of this case, it was learned that his family history was good; that on January 17th of this year he had suffered from a slight attack of facial paralysis, and again on April 2d he had experienced a slight attack of hemiplegia on the right side. From both of these attacks he had rallied, and now nothing was noticeable except a slight deviation of the tongue and weakness in the right leg. Further examination revealed an obscure history of syphilis. The past improvement in his nervous system was due to the use of the iodides in moderate dosage. In view of these facts, a diagnosis of "mixed infection" was made, and he was again put upon specific treatment in largely increased doses.

On the fifth day the abscess ruptured, accompanied with intense fetor, but there was no abatement of the fever or cessation of the cough. In fact, the case seemed rapidly progressive towards a fatal termination. For five days longer, the specific medication, together with symptomatic treatment, was continued, but without perceptible favorable results.

It was then determined to institute the anti-tubercle treatment, and it was commenced on May 25th, with 15 minims of the Paquin "anti-tubercle serum," and continued daily, increasing 5 minims each day until 25 minims were given. At the time of commencing this treatment, the patient was almost exhausted, and in the opinion of several physicians who saw him, his case was considered all but hopeless. After the third day of treatment, there was noticeable a slight reduction in the temperature, and on the fifth day the patient was enabled to secure a comfortable night's rest, the cough having diminished to a great extent, and the exhausting night-sweats having almost entirely ceased.

After the eighth day of treatment, the improvement was still greater, the temperature becoming normal, the appetite returning, and the cough and night sweats almost entirely abating.

The treatment was now continued at 20 minims almost daily, for on two occasions when the dose was gradually increased to 40 minims, an irritative febrile condition was induced, and at the same time an increase of albumen in the urine, and indications of an erythematous eruption were noticed. In two weeks the patient was enabled to sit up for several hours at a time, and was altogether comfortable.

The treatment was continued till July 1st, having consumed a little more than one month, and on the following day the patient walked a mile and stood for four hours in the street, reviewing a parade. He is still under observation, and is now taking ninety drops of a saturated solution of potassium iodide daily.

The happy termination of this case, it is admitted, is an exceptional one, but it is illustrative beyond doubt of the efficacy of the serum treatment, even in some cases of mixed infection, which are not the accepted cases for its exhibition.

Some of the features noted in this case worthy of observation are:

1. The speedy reduction in temperature—having improved after the fifth injection, and becoming normal after the eighth.

2. The improvement in the cough and expectoration, the fetor ceasing entirely after the tenth injection.

3. The abatement of the night-sweats, and their subsequent cessation at the expiration of the second week.

4. The return of the appetite, and a gain up to the present time of eighteen pounds in weight.

5. The improvement in the consolidated lung tissue, though this condition cannot be said yet to be "cured," but the microscope reveals a continually decreasing number of bacil present.

DR. VIRGINIUS W. HARRISON said that the case reported by Dr. Hodges recalled to his mind a case similar in some respects. A white man, aged 32, tubercular history, two deaths in his family, he himself having suffered from what was called scrofula in his early childhood—the scars of the removal of the glands now disfiguring his neck. The patient was then emaciated, appetite gone, night-sweats, and a condition which looked like an early dissolution of the body. The patient said that he had suffered from shortness of breath and cough for several months—the shortness of breath increasing. On examination of his lungs, the right side showed that the apex was doing practically no work; the lower portion was consolidated.

Temperature 102°. Dr. Harrison advised him to go home and go to bed, but he declined to go, and went to work, and continued to work for two days, when he had a chill. On the third day, he had another chill, and went to bed. The doctor saw him on the fourth day, when he said that, after the first chill, he suffered intense pain in the whole right side. Some hours after the second chill, his cough increased a great deal, and soon a bloody, offensive pus was expectorated; during that twenty-four hours he spit out one quart of this material. This continued, so that in the next twenty-four hours he expectorated nearly a pint, and gradually diminished until six weeks had passed and it ceased. Dr. Harrison got a bottle of this material (which he showed to several members of the Academy), and got Mr. Hugh Blair to examine it for him. The examination revealed the nature of the material to be pus, mucus, and blood. Dr. Harrison did not have it examined for the tubercle bacillus, but with such a personal history, and family history, he feels justified in concluding that the condition was due to tuberculosis.

DR. HARRISON remarked that he is aware that tuberculosis of a gland may be perfectly eliminated by removal or suppuration, when the tuberculosis is in the gland alone; and he is further aware that the same bacillus may be latent in other portions of the body, awaiting renewed excitation to renew its energy in its deadly work. This man was treated by inhalations of beechwood creosote, and given liquid peptonoids and tonics. He has recovered sufficiently from this spell to return to his former occupation. The future of this man, in his opinion, is most doubtful, but the advocates of the serum therapy seem unwilling to show their confidence in its use by dropping the drugs they have found in years past to be useful in the same way that serum therapy claims to aid in the disease.

Dr. Harrison added that he would be glad to hear from Dr. Landon B. Edwards on this subject, as we know he has had a good many patients under treatment by this new anti-tubercle serum method.

DR. JAMES N. ELLIS remarked that, in using anti-tubercle serum, it is well to look out for the development of an eruption. He ignored the first red blotches, and the man did not sleep for five days. Injection of three-fourths of a grain of morphine had no effect whatever in allaying the intense irritation.

THE PRESIDENT said that, up to the present time, he had treated fourteen cases with Paquin's anti-tubercle serum. Two of these, referred to him by other physicians, are hopeless. In one of these two, whenever coughing results in free expectoration, the temperature is reduced to 99° or 100°, but in twenty-four hours it is up again. Cavernous respiration is so marked that, as was said, no hope could be held for recovery. The second of the two is even more hopeless. It is a constant matter of wonder how he lives. In three other cases there has been no material improvement, but neither have they grown worse. In the remainder there has been such improvement that two have been discharged apparently cured, but with advice to return occasionally for inspection, as it were. One of these is a vocalist, who says she sings with the same power as before she was afflicted. The second is a medical student, who, since his discharge, has gone through a mild attack of pneumonia, which left some slight consolidation. The hot weather prevents, in a measure, carrying out all details of treatment.

Right here may be remarked that, with this record, it must be acknowledged this treatment is of great benefit.

DR. EDWARDS said he never could have expected such good results from creosote and tonic treatment generally, as he has had in the nine cases which include the two just cited. One of those discharged is using creosote internally. In one case, now much improved, death was expected in three months at most; and while he is not completely cured yet, the advance toward it is remarkable. In another case, the attack was in its incipiency, and creosote alone might have cured without the serum. Whenever the temperature is high, and remains so, no treatment is of service. Edson's treatment has not seemed to make any favorable impression.

The cases mentioned, in which fatal issue seems inevitable, are of mixed infection.

If Paquin's treatment had no curative value, its palliative qualities would alone render it remarkable—stopping night-sweats, non-interference with appetite, if anything, increasing it, etc. In hot weather, cod-liver oil does more harm than good. If employing it, do so in the form of emulsion. When the stomach is enervated, it does as much damage as anything can.

DR. HARRISON: Have you used the serum treatment alone? If not, is it because you have not the necessary confidence?

DR. EDWARDS replied that he employs accessory treatment, simply as in fighting he would employ both fists, not depending on the right solely. With so dangerous an enemy as tuberculosis, he is unwilling to do less than everything that promises good.

DR. HODGES stated he had used the serum treatment alone with benefit.

### ABSCESS OF PALM OF HAND FOLLOWING TYPHOID FEVER.

THE SECRETARY reported the case of a boy convalescing from typhoid fever, who, two or three days after temperature reached normal, complained of pain in the right hand. Next day the pain was more severe, and swelling had occurred. Salicylates were prescribed with the result that, on the third day, pain had subsided, but swelling was found greatly increased. That afternoon information was received that the "hand had burst and corruption was pouring out." On investigation, this was found to be true, and further, that the pus had dissected its way clear to the tips of the fingers and down to the wrist, the whole hand and all the fingers being involved except the thumb and thenar eminence. Incisions were made between the index and middle, and ring and little fingers, to facilitate drainage. Under the daily employment of hydrogen dioxide and carbolized water, pus formation is rapidly ceasing. Of course, thrombus was diagnosed, but the wonder is at the rapid formation of pus.

